# Human endometrial regenerative cells attenuate renal ischemia reperfusion injury in mice

**DOI:** 10.1186/s12967-016-0782-3

**Published:** 2016-01-28

**Authors:** Peng Sun, Jian Liu, Wenwen Li, Xiaoxi Xu, Xiangying Gu, HongYue Li, Hongqiu Han, Caigan Du, Hao Wang

**Affiliations:** Department of General Surgery, Tianjin Medical University General Hospital, 154 Anshan Road, Heping District, Tianjin, 300052 China; Tianjin General Surgery Institute, Tianjin, China; Department of Gastroenterology and Hepatology, Tianjin Medical University General Hospital, Tianjin, China; Department of Gynecology and Obstetrics, Tianjin Medical University General Hospital, Tianjin, China; Department of Urologic Sciences, The University of British Columbia, Vancouver, BC Canada; Immunity and Infection Research Centre, Vancouver Coastal Health Research Institute, Vancouver, BC Canada

**Keywords:** Endometrial regenerative cells, Ischemia–reperfusion injury, Kidney

## Abstract

**Background:**

Endometrial regenerative cells (ERCs) is an attractive novel type of adult mesenchymal stem cells that can be non-invasively obtained from menstrual blood and are easily replicated at a large scale without tumorigenesis. We have previously reported that ERCs exhibit unique immunoregulatory properties in experimental studies in vitro and in vivo. In this study, the protective effects of ERCs on renal ischemia–reperfusion injury (IRI) were examined.

**Methods:**

Renal IRI in C57BL/6 mice was induced by clipping bilateral renal pedicles for 30 min, followed by reperfusion for 48 h. ERCs were isolated from healthy female menstrual blood, and were injected (1 million/mouse, i.v.) into mice 2 h prior to IRI induction. Renal function, pathological and immunohistological changes, cell populations and cytokine profiles were evaluated after 48 h of renal reperfusion.

**Results:**

Here, we showed that as compared to untreated controls, administration of ERCs effectively prevented renal damage after IRI, indicated by better renal function and less pathological changes, which were associated with increased serum levels of IL-4, but decreased levels of TNF-α, IFN-γ and IL-6. Also, ERC-treated mice displayed significantly less splenic and renal CD4^+^ and CD8^+^ T cell populations, while the percentage of splenic CD4^+^CD25^+^ regulatory T cells and infiltrating M2 macrophages in the kidneys were significantly increased in ERC-treated mice.

**Conclusions:**

This study demonstrates that the novel anti-inflammatory and immunoregulatory effects of ERCs are associated with attenuation of renal IRI, suggesting that the unique features of ERCs may make them a promising candidate for cell therapies in the treatment of ischemic acute kidney injury in patients.

## Background

Renal ischemia–reperfusion injury (IRI) is a major cause of clinical acute kidney injury (AKI), which is a frequent and serious problem in transplantation, renal surgery, renal diseases and trauma [1]. Vigorous research on IRI has been conducted, but the mortality rate of AKI remains high. There is no effective therapy available to treat renal IRI or ischemic AKI. Thus, new therapeutic approaches are desperately needed.

The underlying mechanisms of renal IRI are complex and multiple factors are involved. The pathophysiology of renal IRI includes initial cellular damage caused by ischemia, as well as delayed renal injury resulting from inflammatory and immune responses following reperfusion [2]. Numerous studies have demonstrated that renal IRI is an inflammatory disease, which is mediated by innate and adoptive immune responses [3–6]. The activation and infiltration of neutrophils [5, 7], macrophages [8], T lymphocytes [9], monocytes and inflammatory mediators are involved in modulating severity of injury in experimental models of renal IRI [10–13]. Animal models have showed that inflammation begins as early as 30 min of reperfusion, and inhibition of the immune response activated by renal IRI dramatically improves renal function and histological integrity after ischemia [14].

In the past few years, stem cell-based therapy as a promising alternative solution draws considerable attention for disease treatment. As a potential candidate for regenerative medicine, mesenchymal stem cells (MSCs) promote tissue repair [15–17]. In the meantime, MSCs have immunomodulatory, anti-inflammatory properties and are immune privileged [18]. These cells can modulate the function of immune cells by both cell-to-cell interaction and soluble factor secretion [19, 20]. MSCs can be obtained from different tissues including bone marrow and adipose tissue. However, the disadvantages of using these sources, such as the requirement of invasive access method and the cause of related complications, limited their clinical use and so on [21, 22].

In 2007, Meng et al. discovered the endometrial regenerative cells (ERCs), a novel type of adult MSCs, which satisfy the traits of MSCs but overcome the disadvantages of other conventional sources and the fear of karyotypic abnormalities during culture and possibility of oncogenesis [22]. We and others have reported that ERCs effectively prevent critical limb ischemia [21], attenuate ulcerative colitis [23], stroke [24] and burn injury [25] in mouse models. Also, it has been found that these human cells were not rejected in a xenogeneic animal model [21]. However, whether ERCs could be feasible to simultaneously suppress inflammatory and immune responses, and repair the damaged tissue following IRI remains unclear. Thus, the aim of this study was to explore the potential role of ERC therapy in prevention of renal IRI.

## Methods

### Preparation of ERCs for in vivo treatment

ERCs were collected from menstrual blood of women (20–40 years old) after informed consent was obtained. Five milliliters of menstrual blood were collected by urine cup-tubing method in an antibiotic containing solution. Mononuclear cells were separated by standard Ficoll method, and the resultant samples were suspended in Dulbecco’s modified Eagle’s medium (DMEM) high glucose supplemented with 10 % fetal bovine serum (FBS), and split into two 10 cm dishes. After overnight incubation, cultured cells revealed marginal adherence to the tissue culture flask. Cells were cultured with media changing twice a week. At the completion of 2 week culture, an outgrowth of adherent cells with a fibroblast-like morphology was observed [22]. The estimated adherent cell number at the start of culture was approximately 1 × 10^7^ [26].

### Animals

Eight-week-old male C57BL/6 mice (Aoyide Co., Tianjin, China) weighing 20–25 g were housed under conventional experimental environment with 12 h light–dark cycle in the Animal Care Facility of Tianjin General Surgery Institute. The mice had a free access to commercial standard mouse diet and water. All experiments were conducted in accordance with the protocols approved by the Animal Care and Use Committee of Tianjin Medical University (China) according to the Chinese Council on Animal Care guidelines.

### Experimental groups

The experiment was conducted following the protocol described previously [27]. In brief, to test the efficacy of ERCs in attenuation of kidney IRI, C57BL/6 mice were randomly assigned to three groups (n = 6 per group): (1) sham control group, mice were operated by opening and closing the abdomen, without clipping the renal pedicles; (2) untreated IRI group, through a middle abdomen incision, bilateral renal pedicles were clipped for 30 min, then released [27]; and (3) ERC-treated IRI group, 1 × 10^6^ ERCs were intravenously injected 2 h before clipping bilateral renal pedicles [28]. After reperfusion was confirmed, the abdomen was closed in two layers using standard 6-0 sutures. Mice were maintained by continuous monitoring using a temperature-controlled self-regulated heating system after the completion of surgery. To maintain body fluid balance, 1 mL of warm saline was administered intraperitoneally immediately after operation. The mice were sacrificed 48 h after reperfusion for IRI assessment.

### Assessment of renal function

Serum levels of both blood urea nitrogen (BUN) and creatinine (Cr) were measured when the mice were sacrificed 48 h after renal reperfusion using multichannel analyzer in the Tianjin Medical University General Hospital (Tianjin, China) for assessing the renal function.

### Histological examination

Kidney tissues from each mouse in each group were fixed by 10 % buffered formalin, and embedded in paraffin. Paraffin sections of kidneys (5 μm) stained with Hematoxylin and eosin (H&E) were used for morphology analysis under light microscopy. A semi-quantitative score of tubular damage including cellular necrosis, loss of brush border, cast formation, vacuolization, and tubule dilation were counted as follows: 0, none; 1, <10 %; 2, 11–25 %; 3, 26–45 %; 4, 46–75 %; and 5, >76 % [29].

### Immunohistochemistry

To quantitate the cell infiltration, sections were stained with antibodies (Abcam, http://www.abcam.com/). We performed immunohistochemical staining by using anti-CD3 and anti-Ly6G antibodies for detecting intra-renal cellular infiltration of T cells and neutrophils, respectively. The primary antibody was at a dilution of 1:100. Negative control sections, only stained with secondary antibodies but without primary antibodies, were performed to assess the non-specific staining. The specimens were stained according to the instructions of Strept Avidin–Biotin Complex (SABC) kit. Stained sections were photographed using an Olympus inverted microscope (Olympus Imaging America, Center Valley, PA).

### Flow cytometric analysis

Phenotypic analysis of various immune cells was performed using a flow cytometry with staining with antibodies against CD4, CD8, CD25, F4/80 or CD206 (*e*Biosciences, http://www.ebioscience.com/), according to the manufacturer’s instructions. The percentage of each phenotype of immune cells was analyzed with the corresponding Flowjo software.

### ELISA

The levels of TNF-α, IFN-γ, IL-6 or IL-4 in the serum samples taken from mice 48 h after IRI were measured using an ELISA Kit (Biolgend, http://www.biolegend.com/) according to the manufacturer’s instructions.

### Statistical analysis

Statistical analysis was performed in SPSS version 17.0 software (SPSS Inc., Chicago, USA) and the experimental data were presented as mean ± standard derivation (SD). The data obtained from sham control group, untreated IRI group and ERC-treated IRI group were compared using one-way analysis of variance (ANOVA) to study the effects of ERC treatment on the renal function, histological scores, cell populations and cytokine profiles for repeated measurements. The case number for each experiment is specified in the corresponding figure. *p* value (*p)* < 0.05 was considered statistically significant.

## Results

### ERCs protected renal function in mice with renal IRI

**Fig. 1:**
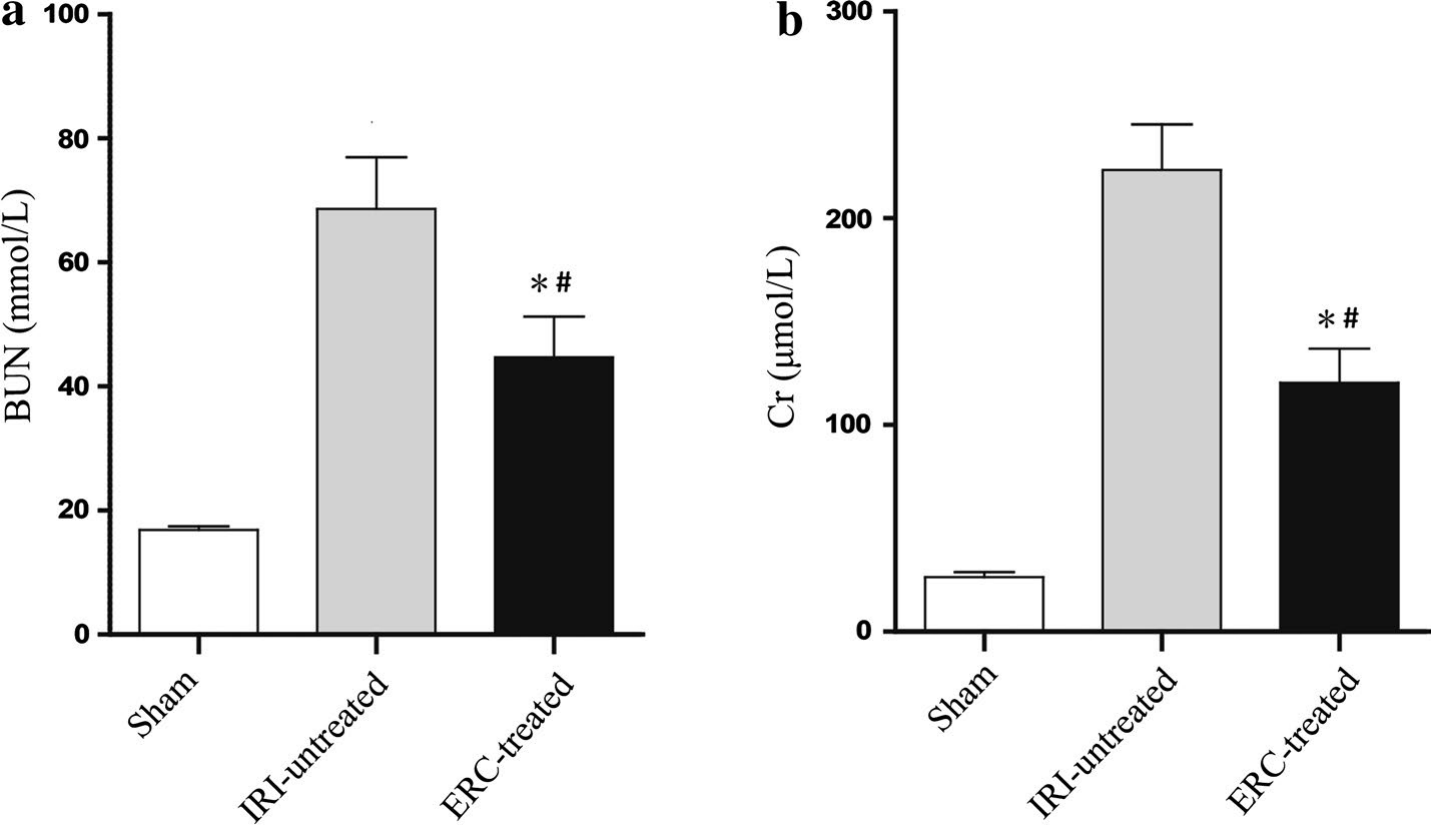
Infused ERCs improves renal function. Serum samples were collected from mice of the sham, IRI-untreated and ERC-treated groups. In comparison with untreated IRI group, infused ERCs significantly reduced serum levels of **a** BUN and **b** Cr. *Bar graphs* represent mean ± SD of three separate experiments. *p* values were determined by one-way ANOVA. Data shown are representative of three separate experiments performed (*p < 0.05, vs. IRI-untreated group, ^#^ *p* < 0.05, vs. sham group, n = 6)

To determine whether the pre-treatment with ERCs could attenuate renal IRI, we subjected mice to bilateral kidney IRI for 30 min. Then the serum BUN and Cr levels were measured after 48 h of induction of IRI. We found that BUN (Fig. 1a) and Cr (Fig. 1b) levels were markedly reduced by ERC treatment compared with those of untreated mice after IRI (*p* < 0.05), although the levels of BUN and Cr were slightly higher than those of mice in sham control group (*p* < 0.05).

### Treatment with ERCs ameliorated renal pathological damages induced by IRI

**Fig. 2:**
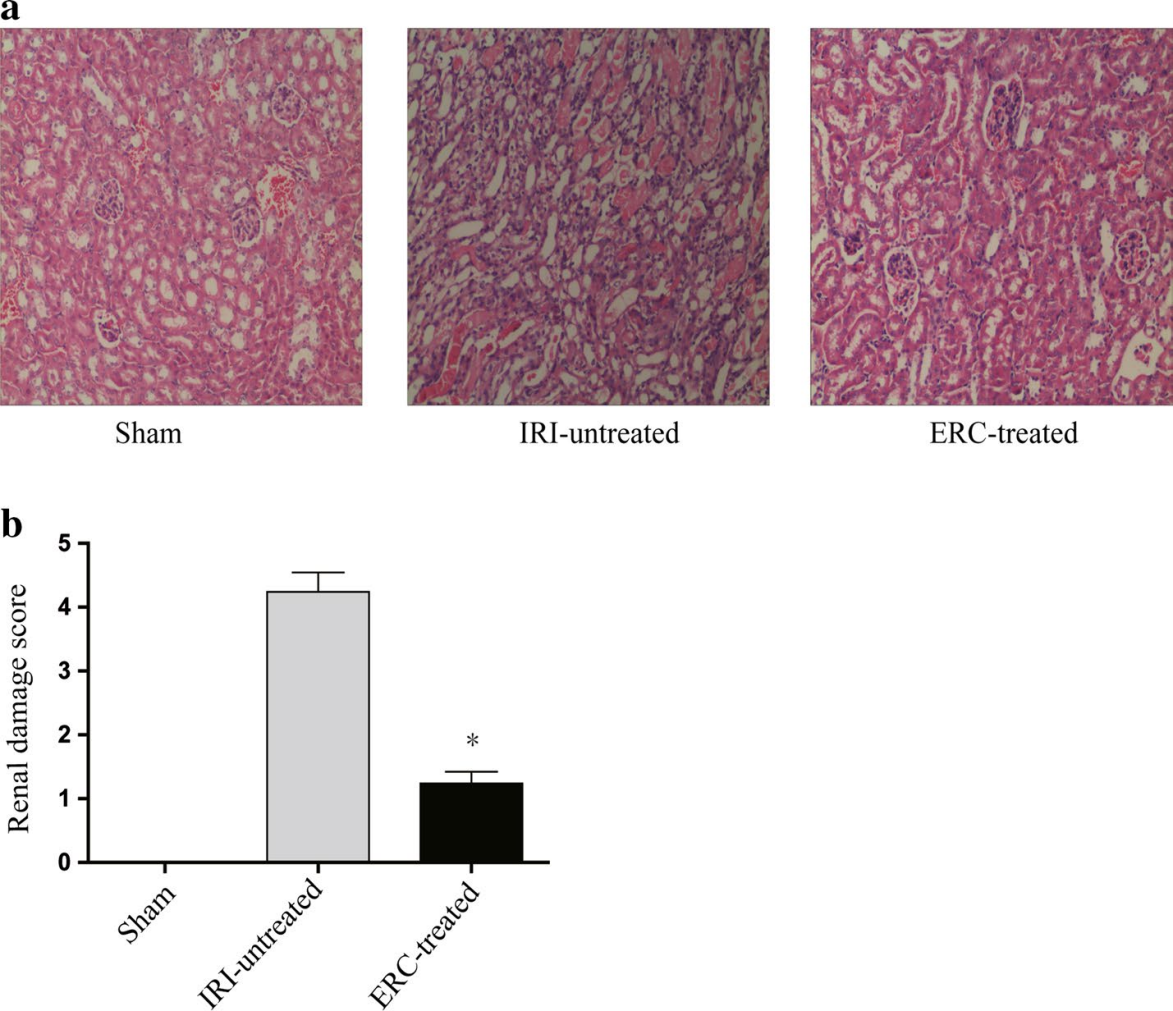
Treatment with ERCs significantly attenuates pathological damage induced by IRI in kidneys. Mouse kidneys were subjected to bilateral renal pedicles clipping for 30 min, then released and followed by 48 h reperfusion. Mice were sacrificed and the kidneys were harvested 48 h after reperfusion. **a** H&E staining was performed to evaluate renal pathological changes. The representative kidney sections of different groups were displayed. Magnification 100×. **b** Quantitative assessment of kidney damage was performed as described in the “Methods” section. *Bar graphs* represent mean ± SD of three separate experiments. *p* values were determined by one-way ANOVA. Data shown are representative of three separate experiments (*p < 0.05, vs. IRI-untreated group, n = 6)

H&E staining was performed to assess the renal pathological changes. Representative kidney sections and the renal damage score were shown in Fig. 2. It was found that untreated IRI mice displayed severe tubular damage 48 h after renal IRI, characterized by widespread tubular necrosis, loss of brush border, cast formation, tubular dilatation, expansion of Bowman’s capsule and interstitial edema (Fig. 2a, IRI-untreated). In contrast, the kidneys in ERC-treated mice showed normal histology (Fig. 2a, ERC-treated) at the same time point, which was indistinguishable from that of sham control mice (Fig. 2a, sham). In addition, the histopathological damage scores in ERC-treated group were significantly lower than those of untreated IRI group (*p* < 0.05), which were comparable to those of sham control group (Fig. 2b).

### ERCs suppressed intra-renal infiltration of neutrophils and T cells after renal IRI

**Fig. 3:**
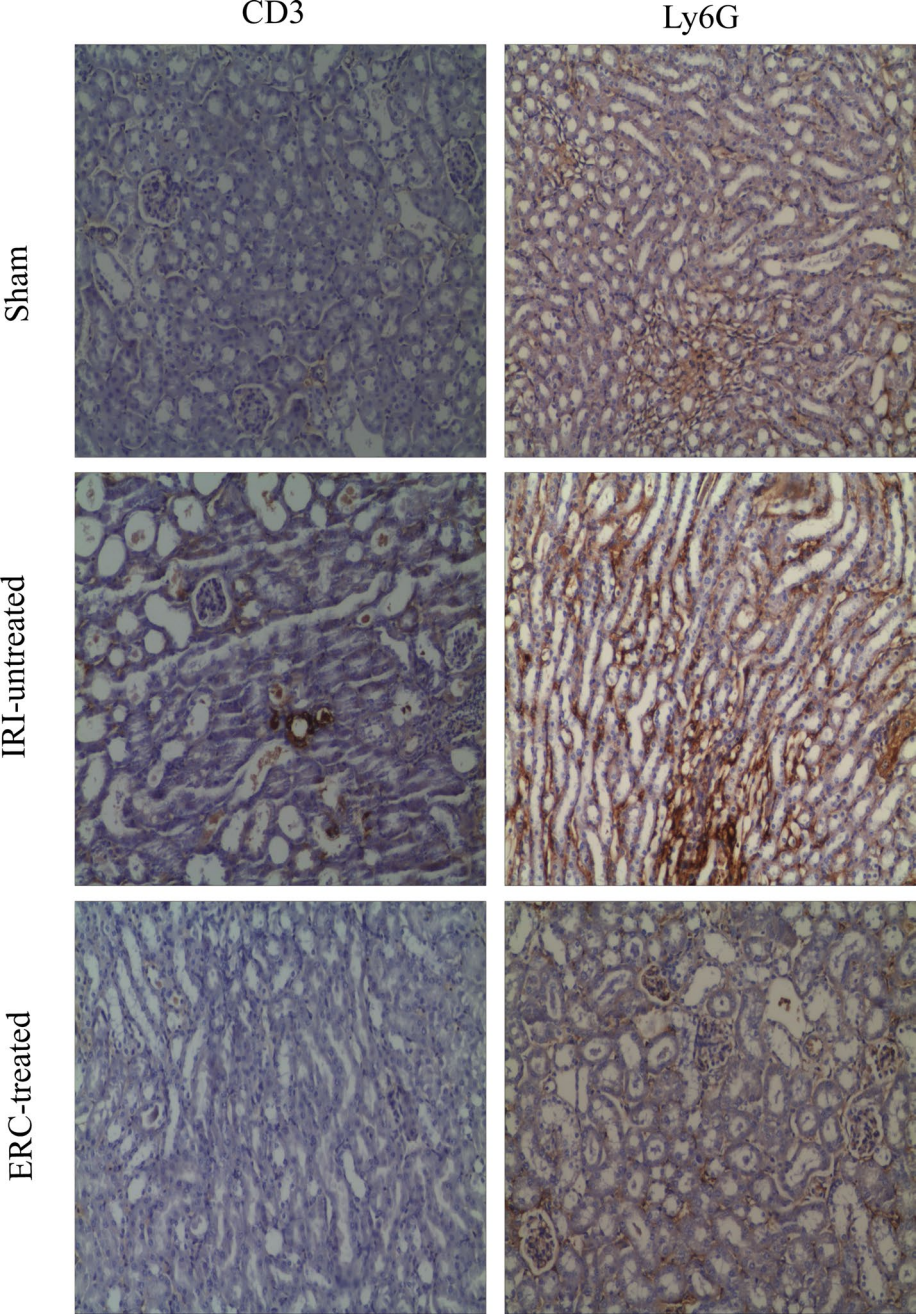
Infused ERCs suppress inflammatory cell infiltration in kidneys in response to IRI. The neutrophil and T cell accumulations in the kidney were examined using anti-Ly6G and anti-CD3 staining respectively 48 h after reperfusion following 30 min-ischemia injury. Representative sections of kidneys were obtained from sham control, IRI-untreated, and ERC-treated IRI mice. Magnification 100×

As shown in Fig. 3, neutrophil and CD3^+^ T cell infiltrations were markedly increased in untreated IRI group as compared with those of sham control group and ERC-treated group. It was suggested that administration of ERCs suppressed neutrophil and CD3^+^ T cell infiltration. As a result, histological injury of kidney was attenuated. Moreover, loss of the brush border, cast formation and tubular dilatation in kidneys were also significantly suppressed in ERC-treated mice.

### Infused ERCs increased the percentage of CD4^+^CD25^+^ T cells in the spleen

**Fig. 4:**
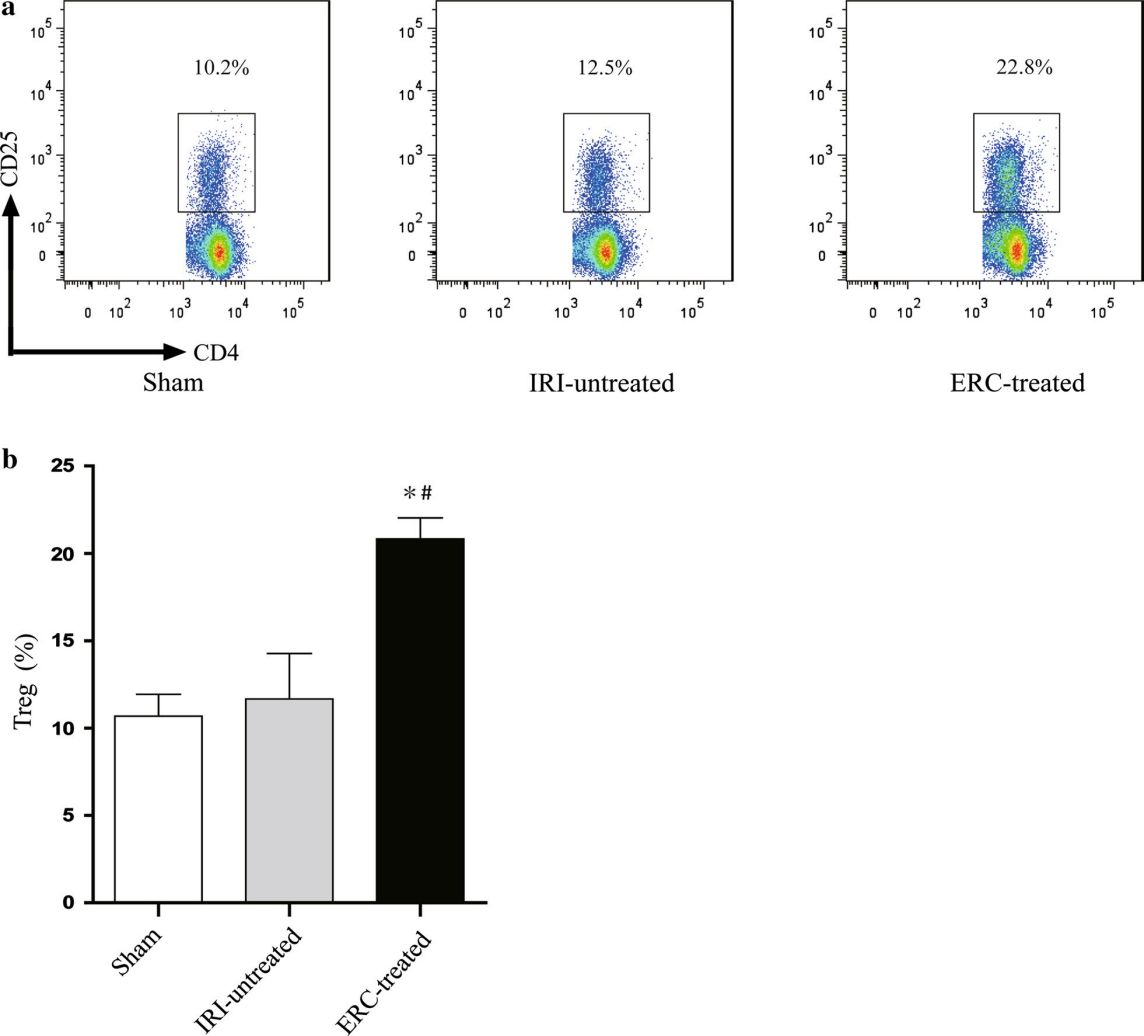
Infused ERCs increase the percentage of CD4^+^CD25^+^ Tregs in the spleen and contribute to the protection of kidney against IRI. **a** Flow cytometric analysis of splenic CD4^+^CD25^+^ Treg population was performed in the sham, IRI-untreated and ERC-treated groups. **b** ERC treatment significantly increased the percentage of Tregs in IRI mice compared with those of untreated IRI mice. *Bar graphs* represent mean ± SD of three separate experiments. *p* values were determined by one-way ANOVA. Data shown are a representative of three separate experiments performed (*p < 0.05, vs. IRI-untreated group, ^#^ *p* < 0.05, vs. sham group, n = 6)

The underlying mechanisms of renal IRI are complex, including activation and progression of immune response. Regulatory T cells (Tregs), playing a critical role in suppression of both adaptive and innate immune responses, have been shown to attenuate renal IRI [28, 30]. In this study, we detected and compared splenic Treg population in different groups. There was no significant difference in the number of Tregs between untreated IRI group and sham control group. In contrast, CD4^+^CD25^+^ Treg population was significantly increased by ERC treatment in mice with renal IRI (*p* < 0.05, vs. untreated IRI group and sham control group), indicating the protective role of Tregs in ERC-mediated kidney protection (Fig. 4).

### ERCs decreased the percentages of CD4^+^ and CD8^+^ T cells in the spleen and kidney after renal IRI

**Fig. 5:**
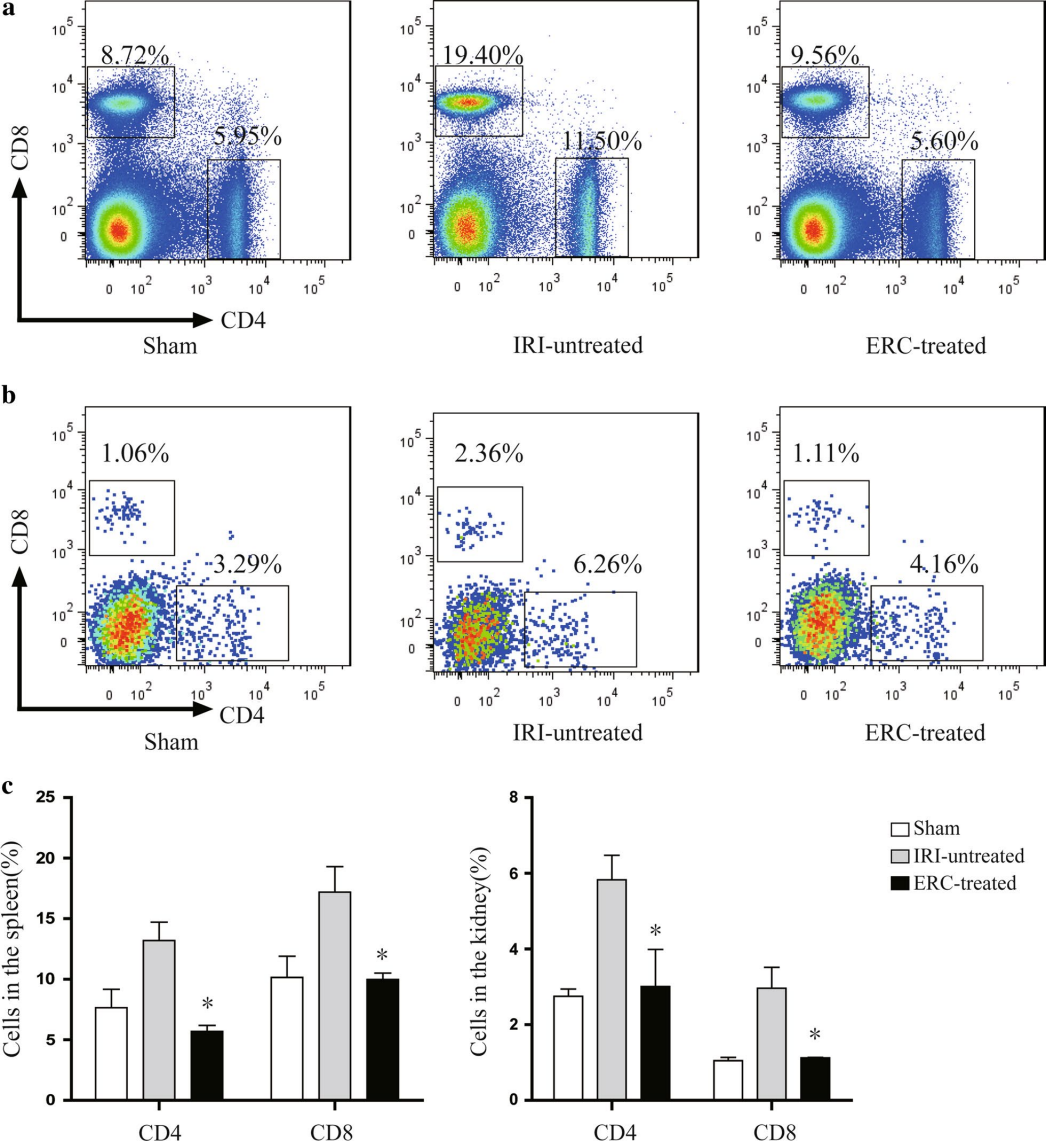
ERCs suppress CD4^+^ and CD8^+^ T cell populations in the spleen and kidney. Flow cytometric analysis of CD4^+^ and CD8^+^ T cell populations was performed in the **a** spleen and **b** kidney of the sham, IRI-untreated and ERC-treated groups. **c** Treatment with ERCs significantly decreased the percentages of CD4^+^ and CD8^+^ T cells in the spleen and kidney. *Bar graphs* represent mean ± SD of three separate experiments. *p* values were determined by one-way ANOVA. Data shown are a representative of three separate experiments performed (*p < 0.01, vs. IRI-untreated group, n = 6)

To determine the relationship between the changes of T cell population and ERC-mediated renal protection, we investigated the infiltration of CD4^+^ and CD8^+^ T cells in both spleen (Fig. 5a) and kidney (Fig. 5b) by flow cytometry. The percentages of CD4^+^ and CD8^+^ T cells were significantly decreased in ERC-treated group compared with those of untreated IRI group in both spleen and kidney (Fig. 5c, *p* < 0.01), which were, in fact, comparable to the control levels in sham control group.

### ERCs suppressed the percentage of total macrophages, but increased M2 macrophages in the kidneys with attenuated IRI

**Fig. 6:**
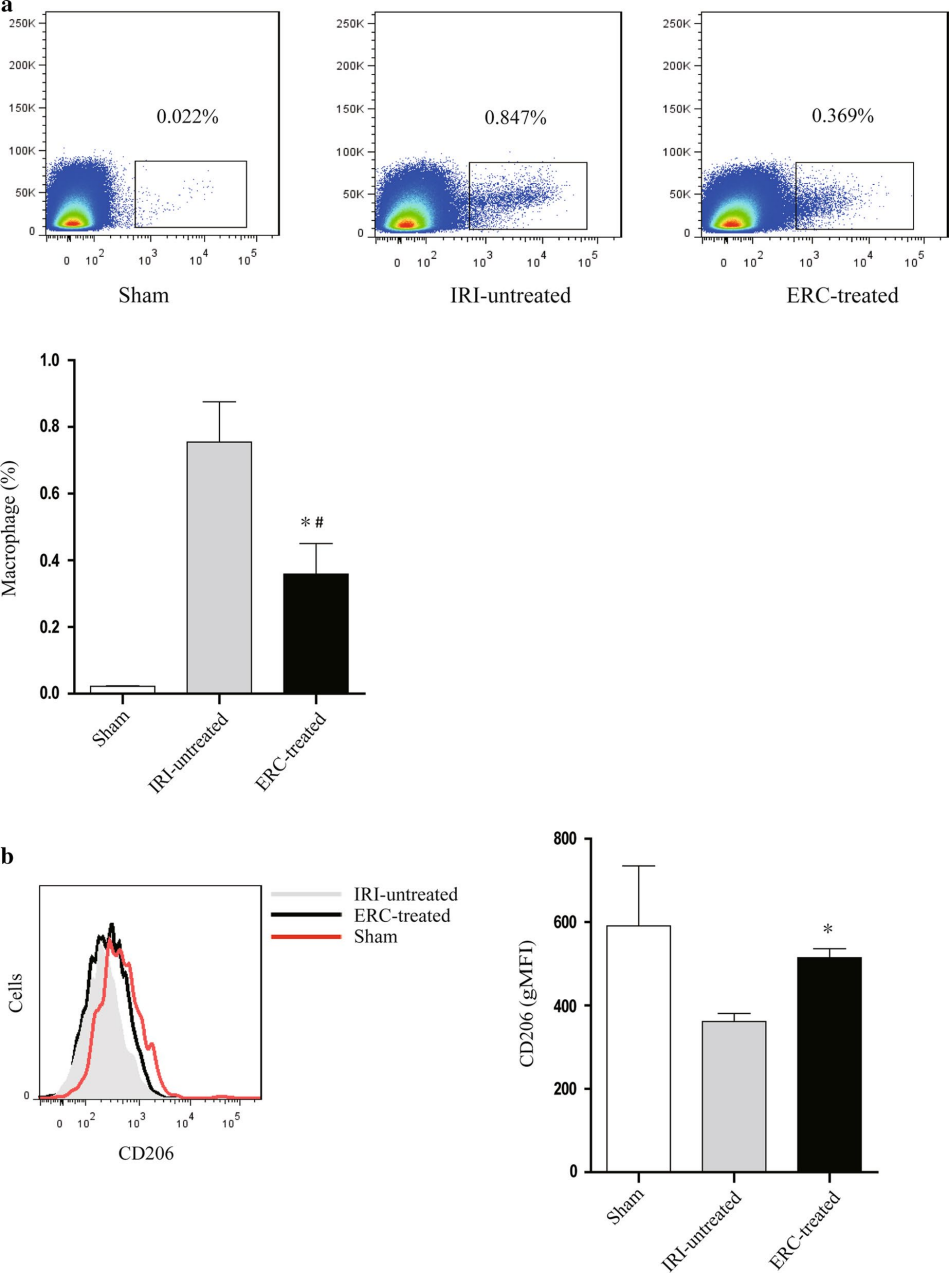
ERCs decrease the percentage of total macrophages but increase M2 macrophages in the kidneys with IRI. Flow cytometric analysis of **a** F4/80^+^ total macrophages and **b** CD206^+^ M2 macrophages was performed in the kidneys of different groups. In Fig. 6a, *vertical axis*: FSC-H, *horizontal axis*: F4/80. *Bar graphs* represent mean ± SD of three separate experiments. *p* values were determined by one-way ANOVA. Data shown are a representative of three separate experiments performed (*p < 0.05, vs. IRI-untreated group, ^#^ *p* < 0.05, vs. sham group, n = 6)

To determine whether the macrophage profile was associated with ERC-mediated attenuation of IRI, we measured total macrophage stained by anti-F4/80 mAb, and M2 macrophages stained by anti-F4/80 and CD206 mAbs by flow cytometry. We found that ERCs effectively suppressed the percentage of total macrophages compared with those of untreated IRI group (*p* < 0.05), even though slightly higher than those of mice in sham control group (*p* < 0.05) (Fig. 6a). Interestingly, M2 macrophage population was significantly increased in ERC-treated mice compared to that of untreated IRI mice (*p* < 0.05). In fact, the percentage of M2 macrophages was undistinguishable between mice in sham control group and ERC-treated IRI group (Fig. 6b).

### ERCs attenuated kidney IRI by regulating cytokine expression

**Fig. 7:**
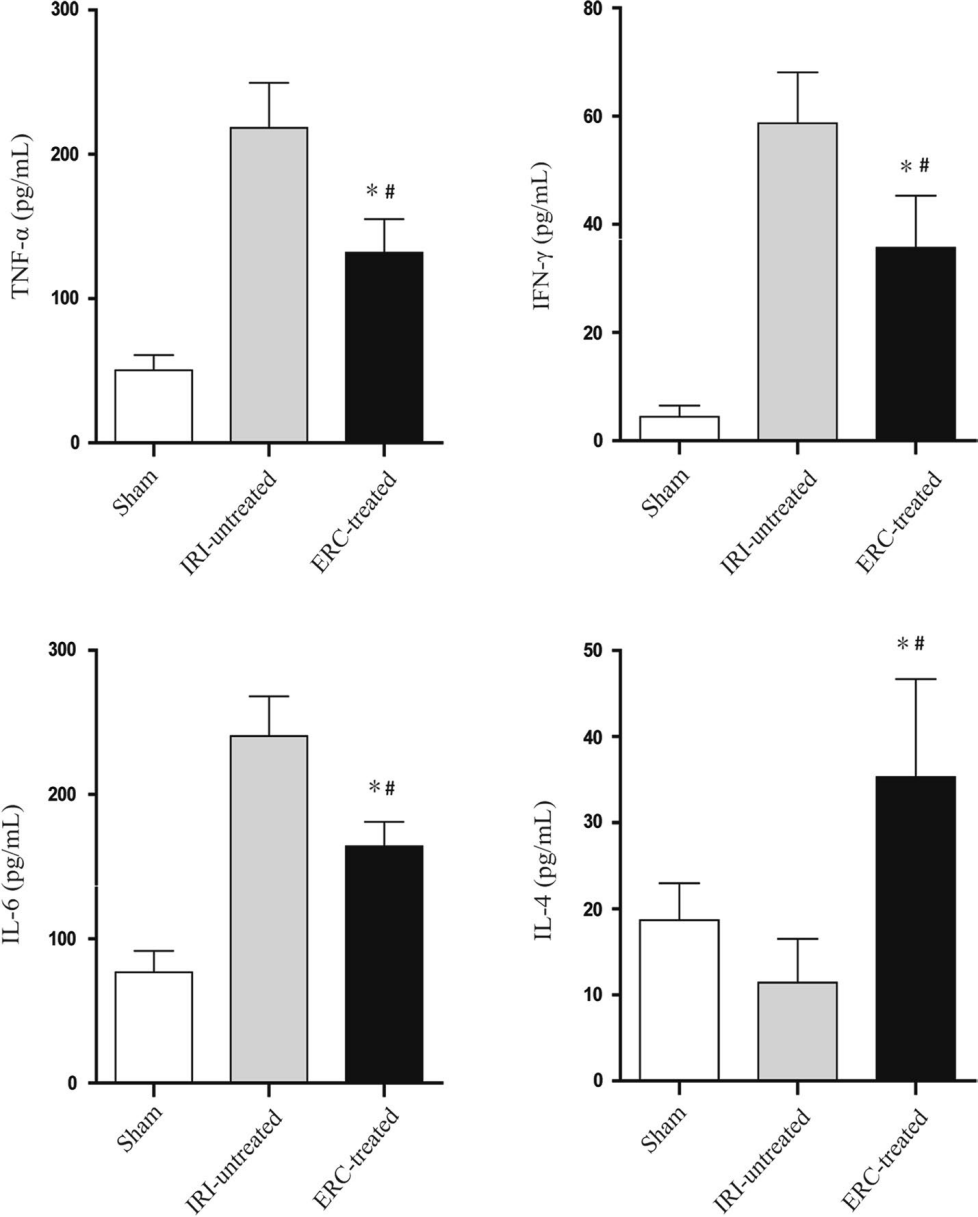
ERCs attenuate renal IRI by regulating cytokine levels. Serum samples were collected from the sham, IRI-untreated and ERC-treated groups. Serum levels of TNF-α, IFN-γ, IL-6 and IL-4 were examined by ELISA. *Bar graphs* represent mean ± SD of three separate experiments. *p* values were determined by one-way ANOVA. Data shown are a representative of three separate experiments performed (*p < 0.05, vs. IRI-untreated group, ^#^ *p* < 0.05, vs. sham group, n = 6)

The serum levels of pro-inflammatory and anti-inflammatory cytokines were analyzed and compared among different groups. As shown in Fig. 7, the serum levels of pro-inflammatory cytokines (TNF-α, IFN-γ and IL-6) were markedly increased in untreated IRI group, whereas ERC treatment significantly reduced the pro-inflammatory cytokine levels in the sera (*p* < 0.05, ERC-treated group *vs*. untreated group), even though slightly higher than the baseline serum levels in sham control group (*p* < 0.05). Meanwhile, treatment with ERCs increased the level of the anti-inflammatory cytokine IL-4 in the sera (*p* < 0.05), which was even higher than that in sham control mice (*p* < 0.05). These data suggest that ERCs not only suppress the expression of pro-inflammatory cytokines, but also enhance the level of anti-inflammatory cytokines.

## Discussion

As we all know, cell therapy has been reported to be an effective method in the treatment of some diseases. ERCs are an attractive novel type of adult MSCs, which can be obtained from menstrual blood. In the current study, we focused on their anti-inflammatory and immunomodulatory properties in attenuation of renal IRI. So we intravenously injected ERCs into mice suffering from renal IRI, an experimental model of ischemic AKI. We have highlighted several findings in this study. First, intravenously injected ERCs decreased intra-renal CD3^+^ T cell and neutrophil infiltration; second, ERCs increased the percentage of splenic Tregs in mice with attenuated renal damage; third, ERCs decreased CD4^+^ and CD8^+^ T cell population in both spleen and kidney; fourth, ERCs down-regulated the percentage of total macrophages, but increased M2 macrophages; finally, injected ERCs suppressed pro-inflammatory cytokines and enhanced anti-inflammatory cytokine expression in mice with ameliorated renal function. To exclude if the therapeutic potential of ERCs is only mediated by cellular component, peripheral blood monocytes were also used as the cell treatment control. As expected, no therapeutic effects were observed according to the assessment of renal function and pathological study (data not shown).

ERCs, an attractive novel type of adult MSCs isolated from menstrual blood, were capable of differentiating into 9 lineages [22]. As we all know, MSCs are a group of self-renewing, pluripotent stromal cells derived from tissues such as bone marrow [31], cord blood [32], adipose tissue [33] and amniotic fluid [34], and have been demonstrated that they could promote tissue repair and possess immunomodulatory and anti-inflammatory properties. ERCs express CD9, CD13, CD14, CD29, CD44, CD73, CD90, CD105, CD117, CD133, CD146 [35], but not STRO-1, CD31 (endothelial) and CD34 (haemopoietic stem cell and endothelial) [36]. The human endometrium has unique immunological requirements in nature. For example, in pregnancy, the endometrium must tolerate invading embryo, which expresses both paternal and maternal antigens. A study demonstrated that endometrial MSCs were able to suppress neural inflammation in a mouse model of multiple sclerosis [37]. MSCs have been demonstrated to inhibit ongoing mixed lymphocyte reaction (MLR) [38], induce generation of regulatory T cells [39], and in vivo suppress autoimmunity such as collagen induced arthritis [40]. But the application of these cell types is limited by availability, invasiveness of extraction and in some cases by proliferative capacity [22]. ERCs are a new type of MSCs, which have some advantages compared with many conventional sources of MSCs. ERCs can be easily expanded and used for females as a non-invasively obtained and ethically appropriate autologous stem cell alternative [22]. We previously reported the role of ERCs in limb ischemia injury [21], UC [23] and recently in prevention of transplant rejection (unpublished data). However the role of ERCs in attenuation of IRI in kidney has not been tested.

The underlying mechanisms of renal IRI are complex, including activation and progression of the immune response [41]. Tregs play a critical role in suppression of both innate and adaptive immune responses [30]. CD4^+^CD25^+^ Tregs account for 5–10 % of the CD4^+^ T cell panel in healthy human and mice, which are sufficient to play an important role in the maintenance of immune homeostasis and the limitation of autoimmune disease [42]. In several studies, the investigators used PC61, an anti-CD25 monoclonal antibody to deplete Tregs before renal ischemia to confirm the protective effect of the Tregs; depletion of Tregs leads to deterioration of the renal IRI compared to those of mice that only suffered from renal IRI and had no other treatment [28, 43, 44]. In another study, the researchers used depletion and adoptive transfer strategy to determine the function of Tregs in the renal IRI model. Administration of anti-CD25 mAb significantly decreased the number of splenic Tregs [28, 45]. In addition, they observed that the adoptive transfer of Tregs back into Treg-depleted mice partially restored the protective effect of ischemic preconditioning [46–48]. Finally, partial depletion and adoptive transfer of Tregs partially mitigated or restored the renal injury [43, 49]. Blocking Tregs worsens AKI and infusion of Tregs protects organs against AKI induced by IRI [46, 50] and cisplatin [51], respectively. Collectively, these data suggested that Treg induction was one of the underlying mechanisms against renal IRI. In current study, we did not find statistically significant difference in the percentage of Tregs between sham control group and untreated IRI group, but observed that Tregs were significantly increased by ERC treatment, even higher than that of sham control group. Thus, it indicated that ERC could increase Tregs which may be related to the suppression of immune response and inflammatory reaction. As a result, intravenous injection of ERCs attenuated renal damage induced by IRI.

To investigate the effects of ERCs on different T lymphocyte subsets, we have also measured the levels of CD4^+^ and CD8^+^ T cells in different groups. Some studies have reported the effects of CD4^+^ and CD8^+^ T cells in renal IRI. In renal IRI, CD4 and CD8 double knockout mice demonstrated significant functional protection [52]. A more direct proof of the role of T cells in IRI has been demonstrated in a mouse liver injury model that a marked protection of liver function was found in T cell-deficient athymic mice [53]. T cell-deficient mice could attenuate renal IRI, and T cell adoptive transfer into these mice restored the injury. Again CD4 knockout mice showed marked protection from renal IRI compared with wild-type mice and adoptive transfer of wild-type CD4^+^ cells into these CD4 knockout mice resulted in a worsening injury [9]. In current study, as compared with untreated IRI group, CD4^+^ and CD8^+^ T cells were significantly reduced after ERC treatment, indicating that ERCs may reduce T cell accumulation. The results suggested that ERCs may have regulatory functions on the cell populations of splenic CD4^+^ and CD8^+^ T cells. All the studies have demonstrated the critical role of CD4^+^ and CD8^+^ T cells in renal IRI.

Macrophages, belonging to the mononuclear phagocyte system, are potent immune regulators which play an important role in tissue homeostasis and remodeling [4, 54–56]. It has been reported that ablation of macrophages by some different strategies 48–72 h after IRI could inhibit tubular repair and enhance injury [57–59]. In another study, adoptive transfer of resting RAW 264.7 macrophages (a mouse leukemic monocyte cell line) in macrophage-depleted mice after IRI could attenuate ischemic AKI and promote kidney repair [53, 60]. Meanwhile, macrophage-cell-based therapy can limit tubular injury and promote renal repair [61]. Accordingly, macrophages show different functions and phenotypes in response to local metabolic and immune microenvironment [62]. In general, there are two broad but distinct subsets of macrophages that are categorized as either classically activated (M1) or alternatively activated (M2) [63, 64]. Recent studies have demonstrated that macrophages are key cells in both the initiation and resolution of renal injury [65–67]. In current study, ERC treatment induced the subset of M2 cells, which may play an important role in attenuation of renal IRI. This is consistent with previous finding that protection of the kidney against ischemia–reperfusion injury and suppression of inflammation could be mediated by inducing M2 polarization [66], as well as the HO-1 expression promotes a “regulatory” M2 phenotype with the large augmentation in IL-10 production [68]. Several studies also have found that the different macrophage subset populations can be recruited and/or activated during different phases of ischemic injury and repair [56, 59, 69]. However, the mechanism of phenotype switching from pro-inflammatory to anti-inflammatory state is not fully understood in damaged kidney caused by IRI. Further studies to dissect these issues are warranted.

In this study, we have also assessed renal inflammatory cytokines 48 h after renal IRI. It is well known that inflammation is induced by IRI, typically occurs in the absence of microorganisms, and has been termed as sterile inflammation [70]. Similar to the response to invading pathogens, the sterile inflammation induced by IRI can also lead to the marked recruitment of neutrophils and the production of cytokines [70]. Neutrophils and macrophages were the initial infiltrates in the injury phase of renal IRI. Similarly, based on our data, IRI also increased the influx of macrophages and neutrophils, but ERCs reduced the influx of those cells during the post-ischemic period. On the other hand, cytokines may play important roles in IRI. Although there is extensive literature regarding the function of cytokines in post-ischemic tissue injury, we only focused on TNF-α, IFN-γ, IL-6 and IL-4 in this study. As assessed by ELISA, the pro-inflammatory cytokines, such as TNF-α, IFN-γ and IL-6, were obviously higher in the untreated IRI group than those of sham group. Once released, they bind with specific receptors to induce the expression of chemokines. These activities promote the recruitment and activation of neutrophils in post-ischemic tissues. Finally, they affected the outcome of the renal IRI.

Concerning the source of ERCs, it has been found that endometrial MSCs’ perivascular location in both the basal and functional layers, indicating that they will be shed each month during menstruation [71]. To date, the source of endometrial MSCs is not yet clear. It is claimed that endometrial MSCs originate from resident stem/progenitor cells. A further study of the specific characterization of ERCs and the mechanisms of ERC-mediated protection in IRI is still underway.

## Conclusions

Our data suggest that ERC treatment is able to protect kidneys against IRI, by significantly decreasing histological damage,inflammatory infiltration, pro-inflammatory cytokine expression, as well as up-regulating Tregs, M2 macrophages and anti-inflammatory cytokines. Therefore, ERCs might provide a novel and potent therapeutic option to prevent renal IRI. The clinical availability of ERCs might further facilitate rapid clinical translation of the present findings in this study.

## Abbreviations

ERC: endometrial regenerative cells
IRI: ischemia–reperfusion injury
Treg: regulatory T cell
MSC: mesenchymal stem cells
TNF: tumor necrosis factor
IL: interleukin

## References

[1] El-Zoghby ZM, Stegall MD, Lager DJ, Kremers WK, Amer H, Gloor JM, Cosio FG (2009). Identifying specific causes of kidney allograft loss. Am J Transplant.

[2] Bonventre JV, Zuk A (2004). Ischemic acute renal failure: an inflammatory disease?. Kidney Int.

[3] Kinsey GR, Li L, Okusa MD (2008). Inflammation in acute kidney injury. Nephron Exp Nephrol.

[4] Jo SK, Sung SA, Cho WY, Go KJ, Kim HK (2006). Macrophages contribute to the initiation of ischaemic acute renal failure in rats. Nephrol Dial Transplant.

[5] Ranganathan PV, Jayakumar C, Mohamed R, Dong Z, Ramesh G (2013). Netrin-1 regulates the inflammatory response of neutrophils and macrophages, and suppresses ischemic acute kidney injury by inhibiting COX-2-mediated PGE2 production. Kidney Int.

[6] Jones DR, Lee HT (2007). Protecting the kidney during critical illness. Curr Opin Anaesthesiol.

[7] Miura M, Fu X, Zhang QW, Remick DG, Fairchild RL (2001). Neutralization of Gro alpha and macrophage inflammatory protein-2 attenuates renal ischemia/reperfusion injury. Am J Pathol.

[8] Day YJ, Huang L, Ye H, Linden J, Okusa MD (2005). Renal ischemia-reperfusion injury and adenosine 2A receptor-mediated tissue protection: role of macrophages. Am J Physiol Renal Physiol.

[9] Burne MJ, Daniels F, El Ghandour A, Mauiyyedi S, Colvin RB, O’Donnell MP, Rabb H (2001). Identification of the CD4(+) T cell as a major pathogenic factor in ischemic acute renal failure. J Clin Invest.

[10] Li L, Huang L, Vergis AL, Ye H, Bajwa A, Narayan V, Strieter RM, Rosin DL, Okusa MD (2010). IL-17 produced by neutrophils regulates IFN-gamma-mediated neutrophil migration in mouse kidney ischemia-reperfusion injury. J Clin Invest.

[11] Li L, Okusa MD (2010). Macrophages, dendritic cells, and kidney ischemia-reperfusion injury. Semin Nephrol.

[12] Jang HR, Rabb H (2009). The innate immune response in ischemic acute kidney injury. Clin Immunol.

[13] Ferenbach DA, Sheldrake TA, Dhaliwal K, Kipari TM, Marson LP, Kluth DC, Hughes J (2012). Macrophage/monocyte depletion by clodronate, but not diphtheria toxin, improves renal ischemia/reperfusion injury in mice. Kidney Int.

[14] Lai LW, Yong KC, Igarashi S, Lien YH (2007). A sphingosine-1-phosphate type 1 receptor agonist inhibits the early T-cell transient following renal ischemia-reperfusion injury. Kidney Int.

[15] Wu Y, Chen L, Scott PG, Tredget EE (2007). Mesenchymal stem cells enhance wound healing through differentiation and angiogenesis. Stem Cells.

[16] Imanishi Y, Saito A, Komoda H, Kitagawa-Sakakida S, Miyagawa S, Kondoh H, Ichikawa H, Sawa Y (2008). Allogenic mesenchymal stem cell transplantation has a therapeutic effect in acute myocardial infarction in rats. J Mol Cell Cardiol.

[17] de Vries DK, Schaapherder AF, Reinders ME (2012). Mesenchymal stromal cells in renal ischemia/reperfusion injury. Front Immunol.

[18] De Miguel MP, Fuentes-Julian S, Blazquez-Martinez A, Pascual CY, Aller MA, Arias J, Arnalich-Montiel F (2012). Immunosuppressive properties of mesenchymal stem cells: advances and applications. Curr Mol Med.

[19] He XW, He XS, Lian L, Wu XJ, Lan P (2012). Systemic infusion of bone marrow-derived mesenchymal stem cells for treatment of experimental colitis in mice. Dig Dis Sci.

[20] Soleymaninejadian E, Pramanik K, Samadian E (2012). Immunomodulatory properties of mesenchymal stem cells: cytokines and factors. Am J Reprod Immunol.

[21] Murphy MP, Wang H, Patel AN, Kambhampati S, Angle N, Chan K, Marleau AM, Pyszniak A, Carrier E, Ichim TE, Riordan NH (2008). Allogeneic endometrial regenerative cells: an “Off the shelf solution” for critical limb ischemia?. J Transl Med.

[22] Meng X, Ichim TE, Zhong J, Rogers A, Yin Z, Jackson J, Wang H, Ge W, Bogin V, Chan KW, Thebaud B, Riordan NH (2007). Endometrial regenerative cells: a novel stem cell population. J Transl Med.

[23] Lv Y, Xu X, Zhang B, Zhou G, Li H, Du C, Han H, Wang H (2014). Endometrial regenerative cells as a novel cell therapy attenuate experimental colitis in mice. J Transl Med.

[24] Borlongan CV, Kaneko Y, Maki M, Yu SJ, Ali M, Allickson JG, Sanberg CD, Kuzmin-Nichols N, Sanberg PR (2010). Menstrual blood cells display stem cell-like phenotypic markers and exert neuroprotection following transplantation in experimental stroke. Stem Cells Dev.

[25] Drago H, Marin GH, Sturla F, Roque G, Martire K, Diaz Aquino V, Lamonega R, Gardiner C, Ichim T, Riordan N, Raimondi JC, Bossi S, Samadikuchaksaraei A, van Leeuwen M, Tau JM, Nunez L, Larsen G, Spretz R, Mansilla E (2010). The next generation of burns treatment: intelligent films and matrix, controlled enzymatic debridement, and adult stem cells. Transplant Proc.

[26] Hida N, Nishiyama N, Miyoshi S, Kira S, Segawa K, Uyama T, Mori T, Miyado K, Ikegami Y, Cui C, Kiyono T, Kyo S, Shimizu T, Okano T, Sakamoto M, Ogawa S, Umezawa A (2008). Novel cardiac precursor-like cells from human menstrual blood-derived mesenchymal cells. Stem Cells.

[27] Wu H, Chen G, Wyburn KR, Yin J, Bertolino P, Eris JM, Alexander SI, Sharland AF, Chadban SJ (2007). TLR4 activation mediates kidney ischemia/reperfusion injury. J Clin Invest.

[28] Hu J, Zhang L, Wang N, Ding R, Cui S, Zhu F, Xie Y, Sun X, Wu D, Hong Q, Li Q, Shi S, Liu X, Chen X (2013). Mesenchymal stem cells attenuate ischemic acute kidney injury by inducing regulatory T cells through splenocyte interactions. Kidney Int.

[29] Wang W, Faubel S, Ljubanovic D, Mitra A, Falk SA, Kim J, Tao Y, Soloviev A, Reznikov LL, Dinarello CA, Schrier RW, Edelstein CL (2005). Endotoxemic acute renal failure is attenuated in caspase-1-deficient mice. Am J Physiol Renal Physiol.

[30] Lee VW, Wang YM, Wang YP, Zheng D, Polhill T, Cao Q, Wu H, Alexander IE, Alexander SI, Harris DC (2008). Regulatory immune cells in kidney disease. Am J Physiol Renal Physiol.

[31] Edwards RG (2004). Stem cells today: B1. Bone marrow stem cells. Reprod Biomed Online.

[32] Harris DT, Badowski M, Ahmad N, Gaballa MA (2007). The potential of cord blood stem cells for use in regenerative medicine. Expert Opin Biol Ther.

[33] Parker AM, Katz AJ (2006). Adipose-derived stem cells for the regeneration of damaged tissues. Expert Opin Biol Ther.

[34] De Coppi P, Bartsch G, Siddiqui MM, Xu T, Santos CC, Perin L, Mostoslavsky G, Serre AC, Snyder EY, Yoo JJ, Furth ME, Soker S, Atala A (2007). Isolation of amniotic stem cell lines with potential for therapy. Nat Biotechnol.

[35] Dominici M, Le Blanc K, Mueller I, Slaper-Cortenbach I, Marini F, Krause D, Deans R, Keating A, Prockop D, Horwitz E (2006). Minimal criteria for defining multipotent mesenchymal stromal cells. The International Society for Cellular Therapy position statement. Cytotherapy.

[36] Dimitrov R, Timeva T, Kyurkchiev D, Stamenova M, Shterev A, Kostova P, Zlatkov V, Kehayov I, Kyurkchiev S (2008). Characterization of clonogenic stromal cells isolated from human endometrium. Reproduction.

[37] Peron JP, Jazedje T, Brandao WN, Perin PM, Maluf M, Evangelista LP, Halpern S, Nisenbaum MG, Czeresnia CE, Zatz M, Camara NO, Rizzo LV (2012). Human endometrial-derived mesenchymal stem cells suppress inflammation in the central nervous system of EAE mice. Stem Cell Rev.

[38] Deng W, Han Q, Liao L, Li C, Ge W, Zhao Z, You S, Deng H, Zhao RC (2004). Allogeneic bone marrow-derived flk-1 + Sca-1- mesenchymal stem cells leads to stable mixed chimerism and donor-specific tolerance. Exp Hematol.

[39] Prevosto C, Zancolli M, Canevali P, Zocchi MR, Poggi A (2007). Generation of CD4 + or CD8 + regulatory T cells upon mesenchymal stem cell-lymphocyte interaction. Haematologica.

[40] Augello A, Tasso R, Negrini SM, Cancedda R, Pennesi G (2007). Cell therapy using allogeneic bone marrow mesenchymal stem cells prevents tissue damage in collagen-induced arthritis. Arthritis Rheum.

[41] Chen GY, Nunez G (2010). Sterile inflammation: sensing and reacting to damage. Nat Rev Immunol.

[42] Himmel ME, Yao Y, Orban PC, Steiner TS, Levings MK (2012). Regulatory T-cell therapy for inflammatory bowel disease: more questions than answers. Immunology.

[43] Lai LW, Yong KC, Lien YH (2012). Pharmacologic recruitment of regulatory T cells as a therapy for ischemic acute kidney injury. Kidney Int.

[44] Rabb H, Ramirez G, Saba SR, Reynolds D, Xu J, Flavell R, Antonia S (1996). Renal ischemic-reperfusion injury in L-selectin-deficient mice. Am J Physiol.

[45] Jun C, Ke W, Qingshu L, Ping L, Jun D, Jie L, Bo C, Su M (2014). Protective effect of CD4(+)CD25(high)CD127(low) regulatory T cells in renal ischemia-reperfusion injury. Cell Immunol.

[46] Kinsey GR, Sharma R, Huang L, Li L, Vergis AL, Ye H, Ju ST, Okusa MD (2009). Regulatory T cells suppress innate immunity in kidney ischemia-reperfusion injury. J Am Soc Nephrol.

[47] Lee H, Nho D, Chung HS, Lee H, Shin MK, Kim SH, Bae H (2010). CD4+CD25+ regulatory T cells attenuate cisplatin-induced nephrotoxicity in mice. Kidney Int.

[48] Kinsey GR, Huang L, Jaworska K, Khutsishvili K, Becker DA, Ye H, Lobo PI, Okusa MD (2012). Autocrine adenosine signaling promotes regulatory T cell-mediated renal protection. J Am Soc Nephrol.

[49] Cho WY, Choi HM, Lee SY, Kim MG, Kim HK, Jo SK (2010). The role of Tregs and CD11c(+) macrophages/dendritic cells in ischemic preconditioning of the kidney. Kidney Int.

[50] Kinsey GR, Huang L, Vergis AL, Li L, Okusa MD (2010). Regulatory T cells contribute to the protective effect of ischemic preconditioning in the kidney. Kidney Int.

[51] Yoshida T, Kurella M, Beato F, Min H, Ingelfinger JR, Stears RL, Swinford RD, Gullans SR, Tang SS (2002). Monitoring changes in gene expression in renal ischemia-reperfusion in the rat. Kidney Int.

[52] Rabb H, Daniels F, O’Donnell M, Haq M, Saba SR, Keane W, Tang WW (2000). Pathophysiological role of T lymphocytes in renal ischemia-reperfusion injury in mice. Am J Physiol Renal Physiol.

[53] Zwacka RM, Zhang Y, Halldorson J, Schlossberg H, Dudus L, Engelhardt JF (1997). CD4(+) T-lymphocytes mediate ischemia/reperfusion-induced inflammatory responses in mouse liver. J Clin Invest.

[54] Ko GJ, Boo CS, Jo SK, Cho WY, Kim HK (2008). Macrophages contribute to the development of renal fibrosis following ischaemia/reperfusion-induced acute kidney injury. Nephrol Dial Transplant.

[55] Wynn TA, Chawla A, Pollard JW (2013). Macrophage biology in development, homeostasis and disease. Nature.

[56] Filardy AA, Pires DR, Nunes MP, Takiya CM, Freire-de-Lima CG, Ribeiro-Gomes FL, DosReis GA (2010). Proinflammatory clearance of apoptotic neutrophils induces an IL-12(low)IL-10(high) regulatory phenotype in macrophages. J Immunol.

[57] Jang HS, Kim J, Park YK, Park KM (2008). Infiltrated macrophages contribute to recovery after ischemic injury but not to ischemic preconditioning in kidneys. Transplantation.

[58] Lin SL, Li B, Rao S, Yeo EJ, Hudson TE, Nowlin BT, Pei H, Chen L, Zheng JJ, Carroll TJ, Pollard JW, McMahon AP, Lang RA, Duffield JS (2010). Macrophage Wnt7b is critical for kidney repair and regeneration. Proc Natl Acad Sci USA.

[59] Lee S, Huen S, Nishio H, Nishio S, Lee HK, Choi BS, Ruhrberg C, Cantley LG (2011). Distinct macrophage phenotypes contribute to kidney injury and repair. J Am Soc Nephrol.

[60] Jung M, Sola A, Hughes J, Kluth DC, Vinuesa E, Vinas JL, Perez-Ladaga A, Hotter G (2012). Infusion of IL-10-expressing cells protects against renal ischemia through induction of lipocalin-2. Kidney Int.

[61] Vinuesa E, Hotter G, Jung M, Herrero-Fresneda I, Torras J, Sola A (2008). Macrophage involvement in the kidney repair phase after ischaemia/reperfusion injury. J Pathol.

[62] Gordon S, Taylor PR (2005). Monocyte and macrophage heterogeneity. Nat Rev Immunol.

[63] Gordon S (2003). Alternative activation of macrophages. Nat Rev Immunol.

[64] Wang Y, Harris DC (2011). Macrophages in renal disease. J Am Soc Nephrol.

[65] Kluth DC, Erwig LP, Rees AJ (2004). Multiple facets of macrophages in renal injury. Kidney Int.

[66] Ranganathan PV, Jayakumar C, Ramesh G (2013). Netrin-1-treated macrophages protect the kidney against ischemia-reperfusion injury and suppress inflammation by inducing M2 polarization. Am J Physiol Renal Physiol.

[67] Huen SC, Cantley LG (2015). Macrophage-mediated injury and repair after ischemic kidney injury. Pediatr Nephrol.

[68] Ferenbach DA, Ramdas V, Spencer N, Marson L, Anegon I, Hughes J, Kluth DC (2010). Macrophages expressing heme oxygenase-1 improve renal function in ischemia/reperfusion injury. Mol Ther.

[69] Alikhan MA, Jones CV, Williams TM, Beckhouse AG, Fletcher AL, Kett MM, Sakkal S, Samuel CS, Ramsay RG, Deane JA, Wells CA, Little MH, Hume DA, Ricardo SD (2011). Colony-stimulating factor-1 promotes kidney growth and repair via alteration of macrophage responses. Am J Pathol.

[70] Kvietys PR, Granger DN (2012). Role of reactive oxygen and nitrogen species in the vascular responses to inflammation. Free Radic Biol Med.

[71] Ulrich D, Muralitharan R, Gargett CE (2013). Toward the use of endometrial and menstrual blood mesenchymal stem cells for cell-based therapies. Expert Opin Biol Ther.

